# A Quasi-experimental Study on the Effect of Pre-entry Tuberculosis Screening for Immigrants on Treatment Outcomes in South Korea: A Difference-in-Differences Analysis

**DOI:** 10.1007/s44197-023-00181-6

**Published:** 2024-01-23

**Authors:** Sarah Yu, Dawoon Jeong, Hee-Yeon Kang, Young Ae Kang, Gyeong In Lee, Hongjo Choi

**Affiliations:** 1 School of Health Policy and Management, College of Health Science, Korea University, Seoul, Republic of Korea; 2 BK21 FOUR R&E Center for Learning Health Systems, Korea University, Seoul, Republic of Korea; 3 Department of Preventive Medicine, Seoul National University, Seoul, Republic of Korea; 4 Department of Cancer Control and Population Health, National Cancer Center Graduate School of Cancer Science and Policy, Goyang, Republic of Korea; 5 Division of Pulmonary and Critical Care Medicine, Department of Internal Medicine, Severance Hospital, Yonsei University College of Medicine, Seoul, Republic of Korea; 6 Institute for Immunology and Immunological Disease, Yonsei University College of Medicine, Seoul, Republic of Korea; 7 The Korean Institute of Tuberculosis, Korean National Tuberculosis Association, Cheongju, Republic of Korea; 8 Department of Preventive Medicine, College of Medicine, Konyang University, Daejeon, Republic of Korea

**Keywords:** Tuberculosis, Immigrant, Program evaluation, Health policy, Vulnerable population

## Abstract

**Objective:**

This study ascertains the effects of the pre-entry tuberculosis (TB) screening policy, which was implemented as a strategy for managing TB among immigrants, on the treatment outcomes of immigrants in South Korea.

**Methods:**

This study linked three different datasets from 2013 to 2018, namely (1) Korean National Tuberculosis Surveillance System; (2) National Health Information Database for patients diagnosed with TB with ICD code A15-A19, B90, or U84.3; and (3) Statistics Korea database related to cause of deaths. To identify the effect of the policy, cohorts comprising Korean and immigrant TB patients notified before (January 1, 2013–December 31, 2015) and after (September 1, 2016–December 31, 2018), the implementations of the policy were established. A difference-in-differences (DID) analysis of the treatment success and mortality rates was performed.

**Results:**

Data from 100,262 TB patients were included in the analysis (before policy implementation: 1240 immigrants and 65,723 Koreans; after policy implementation: 256 immigrants and 33,043 Koreans). The propensity score matching-DID analysis results showed that the difference in the treatment success rate between immigrants and Koreans decreased significantly, from 16% before to 6% after the policy implementation. The difference in the mortality rate between the two groups decreased from − 3% before to − 1% after the policy implementation; however, this difference was insignificant.

**Conclusion:**

The treatment outcomes of immigrant TB patients in South Korea improved after the implementation of the pre-entry active TB screening policy. Future immigrant TB policies should consider establishing active patient support strategies and a healthcare collaboration system between countries.

**Supplementary Information:**

The online version contains supplementary material available at 10.1007/s44197-023-00181-6.

## Introduction

Tuberculosis (TB) remains a significant public health issue not only in low- and middle-income countries with high TB incidence, but also in most high-income countries with a low disease burden [1]. This is because, in an environment that allows freedom of international travel, no country can remain TB-free unless TB is eradicated worldwide [2]. Accordingly, the World Health Organization has been emphasizing government accountability and domestic/international collaboration based on human rights and equity for eradicating TB worldwide [3]. Particularly, immigrants from a high-TB burden country are highly vulnerable to TB in a low-TB burden country [4], indicating the importance of using patient-centered integrative care services between the country of origin and country of arrival to reduce the TB burden in both countries [5].

There are various strategies for reducing TB burden among immigrants. Of these, pre-entry active TB screening is a typical intervention strategy applied before migrating from the country of origin to the country of arrival [6]. Countries that perform pre-entry active TB screening include the United Kingdom (UK), the United States (US), Canada, Austria, France, Israel, Australia, New Zealand, and Jordan [7]. In a study conducted in the US on immigrants from some Asian countries, the rate of active TB patients identified via pre-entry screening accounted for 1.21% (95% confidence interval [CI] 0.85–1.25), which was slightly higher than the 0.31% for the diagnosis by community screening after entry (95% CI 0.11–2.1%) [8]. Despite the reports on some positive outcomes, the effect of pre-entry TB screening for immigrants on the reduction in TB incidences in the country of arrival remains uncertain. Accordingly, the need for additional studies has been suggested [5]. Additionally, while there have been several reports on the effect of the pre-entry TB screening policy on patient identification [9], not many studies have investigated the effect of such a policy on changes in the TB burden and treatment outcomes in the country of arrival.

Meanwhile, South Korea is considered an intermediate TB burden country with approximately 49.4 TB incidences per 100,000 population (as of 2020). Moreover, there has been a steady increase in the percentage of immigrants along with rapid economic growth. TB incidences among immigrants have also been increasing steadily since 2000 [10]. Accordingly, the South Korean government proposed two TB programs specialized for immigrants: pre-entry active TB screening linked to visa application and post-arrival screening linked to visa extension, as well as a pilot program for latent TB screening for immigrants staying in South Korea [11, 12]. Particularly, the pre-entry TB screening policy, which stipulated a mandatory requirement of pre-entry TB screening by chest X-ray for foreigners from countries with a relatively high TB burden (TB incidence: at least 50 per 100,000 population) and who are applying for long- stay (≥ 91 days) visa, was implemented on March 1, 2016. This study aims to ascertain the effects of the pre-entry TB screening policy, which was implemented as a strategy for managing TB among immigrants, on the TB treatment outcomes of immigrants in South Korea.

## Methods

### Study Setting and Population

In this study, the 2013–2018 Korean National Tuberculosis Surveillance System (KNTSS) data, the Statistics Korea database (which reported the cause of deaths in 2013–2020), and patient data from the National Health Information Database for those diagnosed with TB with ICD code A15-A19, B90, or U84.3 were integrated using the resident registration number. After anonymizing the data, we established an immigrant TB patient group as beneficiary and a Korean TB patient group as non-beneficiary before and after policy implementation, respectively. For specific methods of linking the data sources, the methodology used in a previous study was applied [13]. The effective date of implementing the pre-entry TB screening policy was March 1, 2016. Accordingly, the period between before the policy implementation (January 1, 2016) and the first six months after policy implementation (August 31, 2016) was considered the policy transition period. TB patients notified during this period were excluded from the cohorts. Moreover, multidrug-resistant TB patients with *Mycobacterium tuberculosis* resistant to at least rifampicin (RIF) and isoniazid (INH) were also excluded to ensure that the cohorts were established as homogeneous groups.

### Measurement

Immigrants who were directly affected by pre-entry TB screening were defined as those who declared their nationality as “immigrant” in the KNTSS, whereas those who declared “Korean” were defined as native Koreans. The patients were divided into two groups: 1) TB patients notified between January 1, 2013 and December 31, 2015 (before the policy implementation) and 2) TB patients notified between September 1, 2016 and December 31, 2018 (after the policy implementation). Owing to the limitations of using data sources comprising only TB notification and health insurance claims data, this study could not check cure statuses based on bacteriological test results. Alternatively, the number of days of using TB drugs was considered, and treatment success (TS) was defined as cases in which treatment was completed. Treatment completion (TC) was classified and evaluated according to the TB regimen. For a 4-drug regimen (HREZ) with isoniazid (H), rifampicin (R), ethambutol (E), and pyrazinamide (Z), TC was defined as taking the drug for ≥ 144 days, accounting for ≥ 80% of 180 days (6 months). For a 3-drug regimen (HRE), TC was defined as taking the drug for ≥ 216 days, accounting for ≥ 80% of 270 days (9 months). Accordingly, cases that were confirmed as TC based on each regimen were considered TS. Case fatality during treatment was defined as reported cases of mortality within 180 days for HREZ and within 270 days for HRE, regardless of the cause of death. Last, patients who did not belong to either the TS or mortality groups were classified as the treatment non-completion (TNC) group. Ultimately, TB TS rate was defined as the percentage of TC divided by TC + TNC, while TB mortality rate was defined as the percentage of the number of patients who died within two years from the start of treatment among all the TB patients. Regardless of policy, gender (male, female), age (5-year unit interval), disability (none, mild, severe), acid fast bacillus (AFB) smear, AFB culture, TB legions, previous TB history, comorbidities (cancer, end stage renal disease, and diabetes mellitus), and income level were evaluated as covariates that may influence TB treatment outcomes. Household income was categorized into six levels (0–6) with health insurance beneficiaries classified into quintiles (1 = lowest, 5 = highest) and medical aid beneficiaries codified as 0.

### Statistical Analysis

The differences in the distribution of baseline characteristics between Koreans and immigrants were analyzed using Pearson’s chi-square test. To ascertain the effects of the policy on changes in TB treatment outcomes among immigrants, a difference-in-differences (DID) model that shows the differences between the before and after policy implementation periods in the differences between the policy impact (immigrants) and policy non-impact groups (Koreans) was established. To establish similar baseline characteristics for the two groups before the DID analysis, we employed propensity score matching (PSM) as a variable-ratio matching strategy for 1:5 matching with caliper = 0.01 using age, income level, gender, and the TB notification year of immigrants and Koreans. Matching at a ratio of 1:5 can yield higher precision, and consequently, smaller confidence intervals than simple 1:1 matching as 1:5 matching retains more exposed subjects than those in the fixed ratio by not dropping those without the set number of comparison group matches [14, 15]. To ascertain whether the parallel assumption of the DID analysis was satisfied, the yearly differences in the TS and mortality rates of immigrants and Koreans were presented as percentages and a 95% CI. The final DID analysis model was established using a linear probability model, as shown below [16].

$$\mathcal{E}\left(Y|X\right)={\beta }_{0}+{\beta }_{1}T+{\beta }_{2}I+{\beta }_{3}TI+\gamma X+\epsilon ={\text{Pr}}(y=1|x)$$

where *Y* is the measured outcome, which was the binary variable of TC and survival. *T* denotes time (0 for pre-implementation [January 2013–December 2015] and 1 for post-implementation [September 2016 to December 2018]). I denotes intervention (Koreans = 0, immigrants = 1) and *X* represents adjustment variables including gender, age, disability, income level, AFB smear, TB lesions, previous TB history, and comorbidities (cancer, end stage renal disease, and diabetes mellitus). We added two sensitivity analyses: (1) immediate policy effect without transition period and (2) 1-year delayed policy effect with 1-year transition period. We used SAS Enterprise Guide V.7.1 (SAS Institute Inc., Cary, NC, USA) and Stata/MP version 18 (StataCorp LLC, College Station, TX, USA) for all statistical analyses.

## Results

**Fig. 1:**
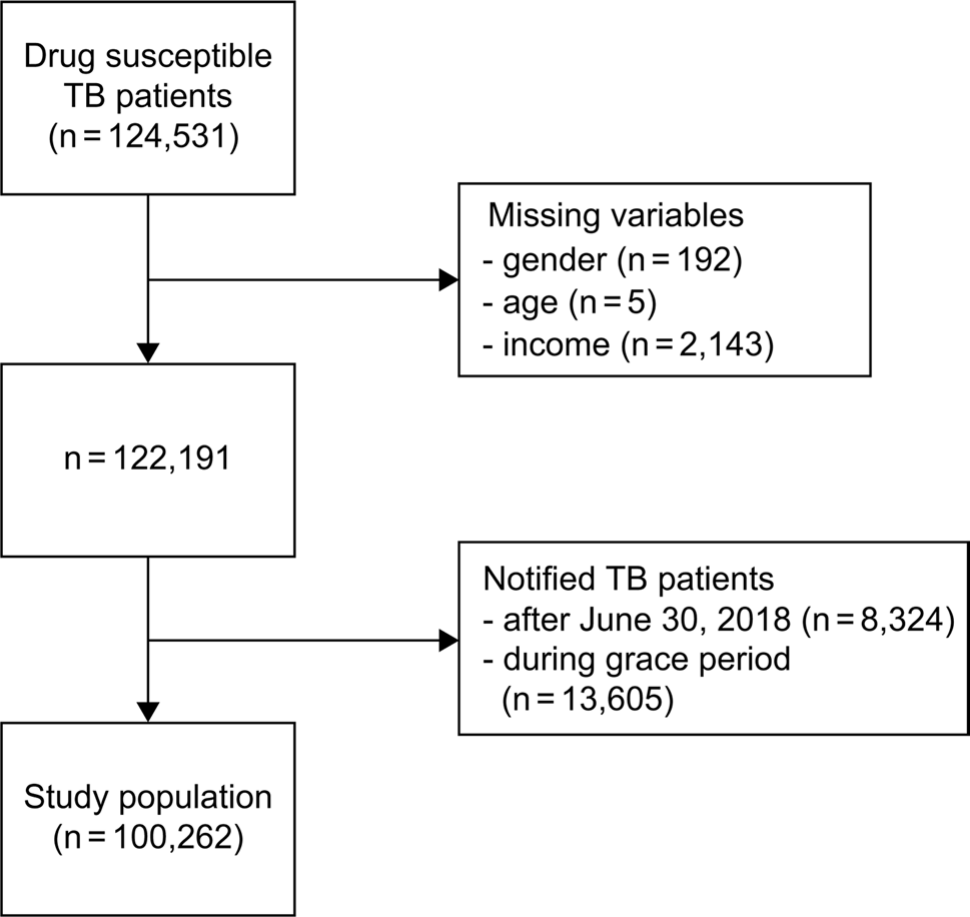
Flow diagram of the study population

**Fig. 2:**
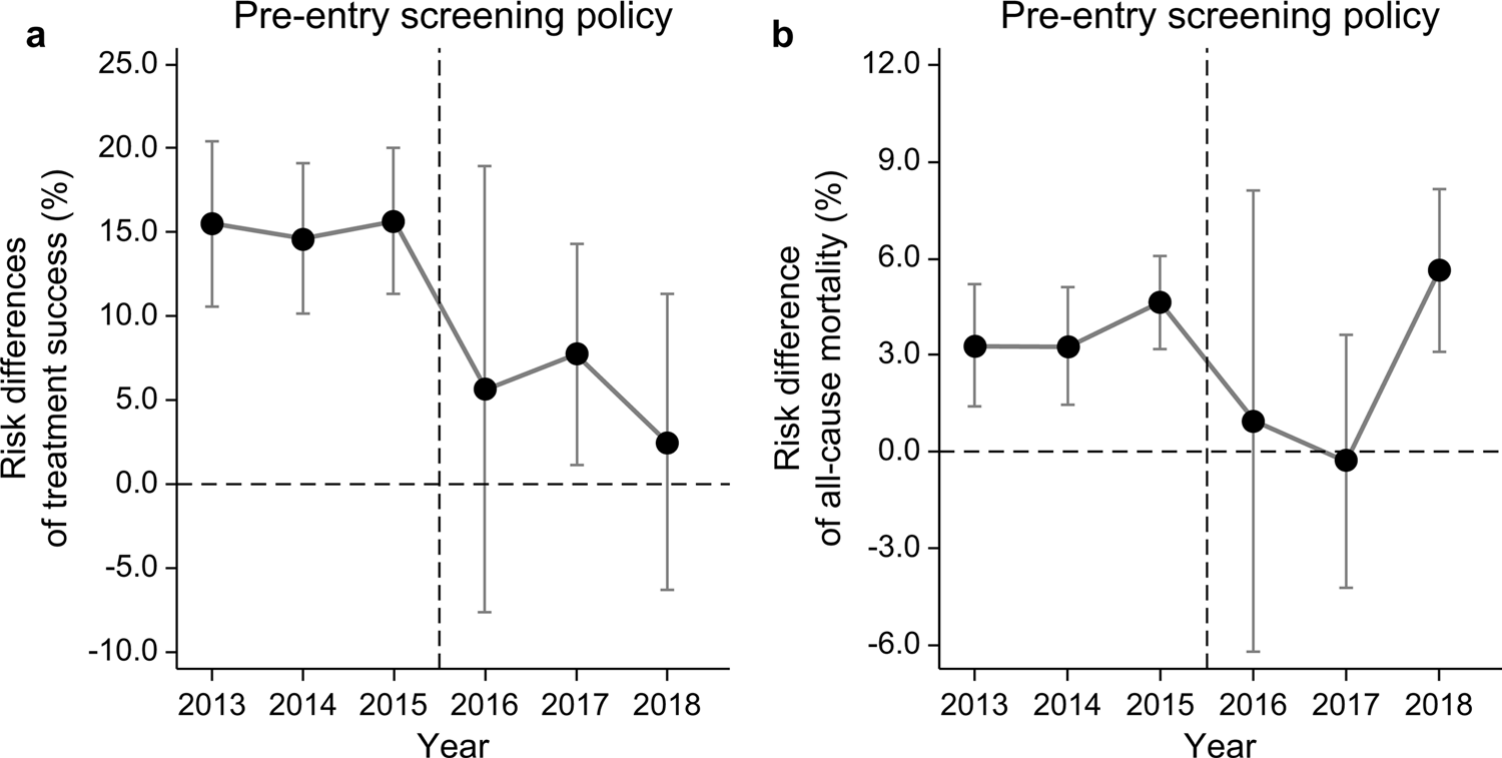
Risk differences in tuberculosis treatment outcomes before and after the pre-entry screening policy **a** treatment success **b** all-cause mortality

Among the 124,531 individuals included in the cohorts during the study period, those with missing values for some variables and cases notified during the policy transition period were excluded. Ultimately, 100,262 individuals (before policy implementation: 1240 immigrants and 65,723 Koreans; after policy implementation: 256 immigrants and 33,043 Koreans) were included in the analysis (Fig. 1). Compared with Koreans, before the PSM, immigrants were relatively younger and had a higher percentage of females, lower household income, and lower notification rate after 2016 (Table 1). Such differences were eliminated after the PSM (Online Resource 1: Table S1). The analysis after the PSM included a total of 8976 individuals (1496 immigrants and 7480 Koreans). Regarding the TS rate of immigrants and Koreans yearly, the difference ranged between 14.6% (95% CI 10.2 ~ 19.1% in 2014) and 15.7% (95% CI 11.4 ~ 20.0% in 2015) before the policy implementation, which decreased to between 2.5% (95% CI − 6.3 ~ 11.2% in 2018) and 7.8% (95% CI 1.2 ~ 14.3% in 2017) after the policy implementation (Online Resource 2: Table S2). Regarding the mortality rate of immigrants and Koreans yearly, the difference ranged between 3.3% (95% CI 1.4 ~ 5.2% in 2013) and 4.6% (95% CI 3.2 ~ 6.1% in 2015) before the policy implementation and between − 0.3% (95% CI − 4.2 ~ 3.7% in 2017) and 5.6% (95% CI: 3.1 ~ 8.1% in 2018) after the policy implementation (Online Resource 2: Table S2). When the changes in the differences in the TS rates of the Korean and immigrant TB patient cohorts between the before and after the implementation periods of the pre-entry active TB screening policy were analyzed via the DID analysis, the results showed that the difference in the TS rates between the Korean and immigrant TB patient cohorts before the policy implementation accounted for 15%, which decreased statistically significantly to 6% after the policy implementation (Table 2). In the covariate-adjusted DID analysis, the results also showed a difference of 16% in the TS rates between the Korean and immigrant cohorts before the policy implementation, which decreased statistically significantly to 6% after the policy implementation (Table 2 and Fig. S1). When the changes in the differences in the mortality rates of the Korean and immigrant TB patient cohorts between the before and after policy implementation periods were analyzed via the DID analysis (Fig. 2), the results showed that the difference in the mortality rates between the Korean and immigrant TB patient cohorts was -4% before the policy implementation and − 1% after the policy implementation, showing no statistically significant difference (Table 3). The covariate-adjusted DID analysis results also showed an insignificant difference in the mortality rates between the cohorts, with values of − 3% before the policy implementation and -1% after the policy implementation (Table 3 and Fig. S2).

**Table 1:** Characteristics of study population before propensity score matching (*n* = 100,262)

	Korean (*n* = 98,766)		Immigrants (*n* = 1496)		
	*N*	%	*n*	%	*p* value
Age group					
~ 14	507	0.51	3	0.2	< 0.001
15–19	2722	2.76	17	1.14	
20–24	3876	3.92	116	7.75	
25–29	4111	4.16	274	18.32	
30–34	4508	4.56	182	12.17	
35–39	4927	4.99	85	5.68	
40–44	6322	6.4	112	7.49	
45–49	7435	7.53	121	8.09	
50–54	8930	9.04	156	10.43	
55–59	9853	9.98	165	11.03	
60–64	7931	8.03	113	7.55	
65–69	6890	6.98	59	3.94	
70–74	8234	8.34	28	1.87	
75 ~	22,520	22.8	65	4.34	
Gender					
Male	58,967	59.7	825	55.15	< 0.001
Female	39,799	40.3	671	44.85	
Disability					
None	85,243	86.31	1488	99.47	< 0.001
Mild	7393	7.49	2	0.13	
Severe	6130	6.21	6	0.4	
Household income					
Medical Aid	9937	10.06	3	0.2	< 0.001
1st quintile (lowest)	16,194	16.4	312	20.86	
2nd quintile	14,832	15.02	399	26.67	
3rd quintile	15,855	16.05	616	41.18	
4th quintile	18,234	18.46	118	7.89	
5th quintile (highest)	23,714	24.01	48	3.21	
Notification year					
2013	23,839	24.14	370	24.73	< 0.001
2014	21,782	22.05	422	28.21	
2015	20,102	20.35	448	29.95	
2016	6317	6.4	42	2.81	
2017	18,361	18.59	150	10.03	
2018	8365	8.47	64	4.28	
Types of notification institution					
Public	1357	1.37	20	1.34	0.903
Private	97,409	98.63	1476	98.66	
Smear test					
Negative	68,613	69.47	1124	75.13	< 0.001
Positive	30,153	30.53	372	24.87	
History of TB					
New case	81,510	82.53	1292	86.36	< 0.001
Relapse	17,256	17.47	204	13.64	
TB classification					
Pulmonary	78,609	79.59	1148	76.74	0.007
Extra pulmonary	20,157	20.41	348	23.26	
Comorbidities					
Malignancy					
No	96,832	98.04	1477	98.73	0.056
Yes	1934	1.96	19	1.27	
Kidney failure					
No	97,661	98.88	1492	99.73	0.002
Yes	1105	1.12	4	0.27	
Diabetes mellitus					
No	75,928	76.88	1402	93.72	< 0.001
Yes	22,838	23.12	94	6.28

**Table 2:** Tuberculosis treatment success rate of Koreans and immigrants before and after the pre-entry tuberculosis screening

	Crude analysis			Adjusted model^a^		
	Coefficient	SE	*p* value	Coefficient	SE	*p* value
Before policy						
Korean	0.88			0.50		
Immigrants	0.72			0.35		
Difference	− 0.15	0.01	< 0.01	− 0.16	0.01	< 0.01
After policy						
Korean	0.90			0.52		
Immigrants	0.83			0.46		
Difference	− 0.06	0.02	0.01	− 0.06	0.02	< 0.01
Difference-in-differences	0.09	0.03	< 0.01	0.10	0.03	< 0.01

^a^Covariates: gender, age, disability, household income, type of notification institution, result of smear, type of tuberculosis, history of TB, comorbidities (malignancy, kidney failure, diabetes mellitus)

**Table 3:** Mortality rate of Korean and immigrant tuberculosis patients before and after the pre-entry tuberculosis screening

	Crude analysis			Adjusted model^a^		
	Coefficient	SE	*p* value	Coefficient	SE	*p* value
Before policy						
Korean	0.06			− 0.07		
Immigrants	0.02			− 0.11		
Difference	-0.04	0.01	< 0.01	− 0.03	0.01	< 0.01
After policy						
Korean	0.05			− 0.08		
Immigrants	0.04			− 0.09		
Difference	− 0.01	0.02	0.36	− 0.01	0.01	0.65
Difference-in-differences	0.02	0.02	0.16	0.03	0.02	0.11

^a^Covariates: gender, age, disability, household income, type of notification institution, result of smear, type of tuberculosis, history of TB, comorbidities (malignancy, kidney failure, diabetes mellitus)

## Discussion

The study findings showed that after the implementation of the pre-entry active TB screening policy, the TS rate of immigrants in South Korea increased by approximately 10% more than that of Koreans. However, the differences in the mortality rate could not be confirmed. Some previous studies investigated and reported the positive effect of pre-entry TB screening using chest X-ray [17]. An Israeli study on Ethiopian immigrants reported that the minimum number of screening personnel required to identify one active TB patient was 291, which was high in both cost-saving effect and efficiency [18]. However, studies that examine whether TB screening for immigrants also affects improvement in treatment outcomes are rare. Most studies that investigate the TB treatment outcomes of immigrants explore the effects of patient care interventions. One Israeli study stated that the treatment outcomes of immigrant TB patients in Israel after the implementation of the directly observed treatment, short-course (DOTS) strategy, which consists of five elements including a directly observed treatment (DOT), improved [19]. Another related study suggested the possibility of lowering the TB mortality rate and reducing related costs in the US by transitioning to a strategy that includes DOT in TB screening for immigrants [20]. Our study points out a major difference in contrast with previous studies, stating that it is possible to improve the TB treatment outcomes of immigrants by reinforcing pre-entry TB screening without altering the patient management strategy.

The observed enhancement in treatment success rate (TSR) can be elucidated by alterations in migrants’ characteristics stemming from TB screening. First, a comparison between the migrant and native Korean populations revealed lower sputum smear positivity rates among migrants, suggesting potentially differing levels of severity or early diagnostic stages (refer to Table 1 and Supplementary Table 1). Additionally, the healthy migrant effect is noteworthy; pre-entry screening might prompt the selective entry of healthier migrants into South Korea. Therefore, the lack of discernible policy impact on TB mortality warrants careful interpretation. TB incidence and mortality rates embody distinct facets of TB management and therapy. While modest policy interventions can influence TSR trends, broader and more stringent policies could substantially impact mortality rates. Pre-entry screening primarily emphasizes the early detection of TB cases. Conversely, addressing TB mortality demands supplementary policies, such as social protection measures tailored for vulnerable migrant populations [5].

To eradicate TB globally, active management for immigrants, who are considered vulnerable to TB, is necessary [21]. In theory, TB treatment outcomes of immigrants could be improved by making hospitals where patients can receive appropriate healthcare services—such as screening, diagnosis, and treatment—accessible, and simultaneously reinforcing the patient care system that includes social protection [22]. Therefore, pre-entry TB screening must be linked to the continuity of actual patient identification and treatment, which must also coincide with investment to reinforce the TB diagnosis and treatment capabilities of the countries of origin. By reinforcing the management of TB patients in the country of origin, the benefits from the screening policy could proportionally aid both countries [23].

Additionally, social protection for immigrants in the country of arrival—enrollment in the social security system, adequate housing provision, and guaranteed access to education and labor market—could be a significant intervention for preventing the latent TB infection of immigrants from progressing to active TB after entering the country of arrival [5]. In our study, the difference in the TB mortality rate of Koreans, which was higher than that of immigrants, disappeared after the policy implementation (risk difference: 1%, 95% CI − 6.2%, 8.1% in 2016). Additionally, the DID analysis results did not show evidence of pre-entry TB screening policy affecting the difference in the TB mortality rates of immigrants and Koreans. While it is difficult to draw any conclusion based on such findings, improving the treatment outcomes from the TB screening policy for immigrants without stronger patient care policies may only be effective for the TS rate, which is a short-term indicator, but may not affect the mortality rate, which is a long-term indicator. The reinforced policy for pre-entry TB screening may aid the early detection of TB among new in-coming immigrants. However, it may not be effective in preventing latent TB from progressing to active TB among immigrants who are already staying in the country. Additionally, the route by which immigrants with latent TB at the time of pre-entry TB screening develop active TB after entering the country of arrival should not be ignored. Delayed diagnosis and inadequate patient care systems during this process could result in a higher mortality rate. Consequently, the difference in the mortality rates between Koreans and immigrants may disappear, as shown in our study. Accordingly, our findings provide some support for international recommendations regarding managing latent TB in migrants and linking them to the TB care system [23–25].

This study has a few limitations. First, because the cohorts were established by linking different data sources, some unidentifiable cases may have been excluded in the process. Consequently, immigrant TB patients who had changed their residence status or those who had settled illegally, such as undocumented aliens, may have been omitted. Therefore, results for immigrants who are relatively more vulnerable may have been excluded, resulting in TS and mortality rates appearing to be slightly higher. Accordingly, the results need to be interpreted with caution.

Second, the length of stay of the immigrants included in our study was not considered. The effect of pre-entry TB screening is likely to be limited to new in-coming immigrants. However, the time of entry of the immigrants in our study could not be determined owing to the limitations of the data used. Approximately 35.2% of foreigners living in South Korea have been in the country for more than three years [26]; therefore, some of the long-term migrants may be included in the study. However, two points would reduce selection bias. First, the policy was initiated with a package of a visa renewal program that must include CXR results for TB diagnosis. Second, most progressions to active TB among migrants occurred within one to three years after they entered, according to previous studies [5, 27].

In conclusion, the study findings confirmed the short-term effect of pre-entry TB screening for immigrants on the improvement in treatment outcomes. For sustained and long-term reduction in TB burden, future immigrant TB policies should consider active patient support strategies throughout the length of stay and establish a healthcare collaboration system between countries.

### Supplementary Information

Below is the link to the electronic supplementary material.

Supplementary Fig 1. Event studies of difference-in-differences over time in tuberculosis treatment success rate (TIF 159 KB)

Supplementary Fig 2. Event studies of difference-in-differences over time in all-cause mortality rate (TIF 168 KB)

Supplementary file3 (DOCX 39 KB)

## Data Availability

The data cannot be shared publicly because of the regulations of the ‘National Health Insurance Service’. Data are available from the Review Board of the National Health Insurance Service (contact via NHIS) for researchers who meet the criteria for access to confidential data. Applications for data are available through National Health Insurance Data Sharing website (https://nhiss.nhis.or.kr/bd/ab/bdaba000eng.do), and additional information can be found at a customized health information data webpage (https://nhiss.nhis.or.kr/bd/ab/bdaba032eng.do).

## Abbreviations

TB: Tuberculosis
DID: Difference-in-differences
KNTSS: Korean National Tuberculosis Surveillance System
RIF: Rifampicin
INH: Isoniazid
TS: Treatment success
TC: Treatment completion
TNC: Treatment non-completion
AFB: Acid fast bacillus
PSM: Propensity score matching
TSR: Treatment success rate
CXR: Chest X-ray
