# Supplementary figures and images for: A Quasi-experimental Study on the Effect of Pre-entry Tuberculosis Screening for Immigrants on Treatment Outcomes in South Korea: A Difference-in-Differences Analysis
Source: J Epidemiol Glob Health. 2024 Jan 23;14(1):154–61. doi: 10.1007/s44197-023-00181-6 (PMC11043236; doi:10.1007/s44197-023-00181-6)

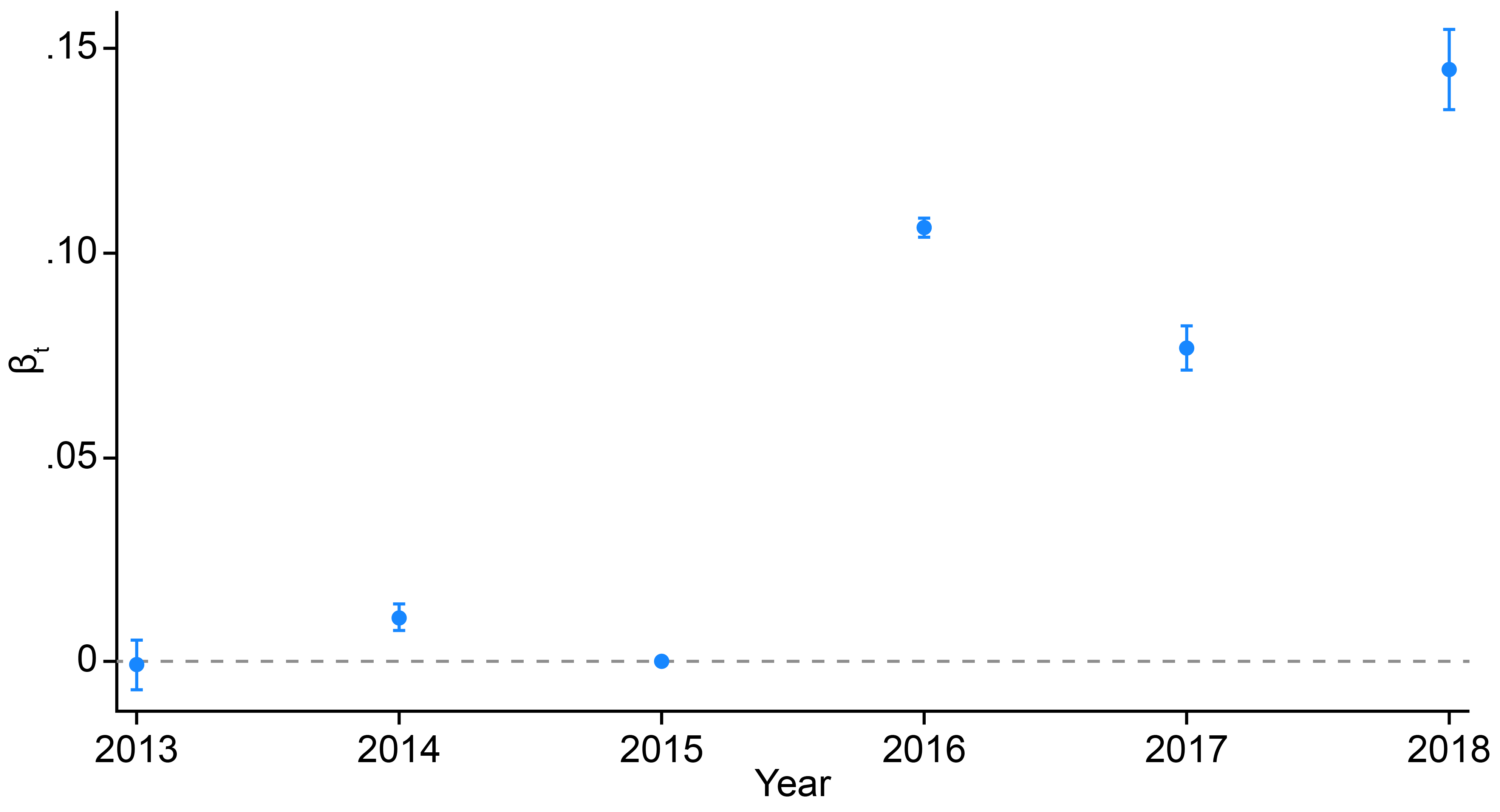

Supplement: Supplementary file 1 — Supplementary Fig 1. Event studies of difference-in-differences over time in tuberculosis treatment success rate (TIF 159 KB) [file 44197_2023_181_MOESM1_ESM.tif]

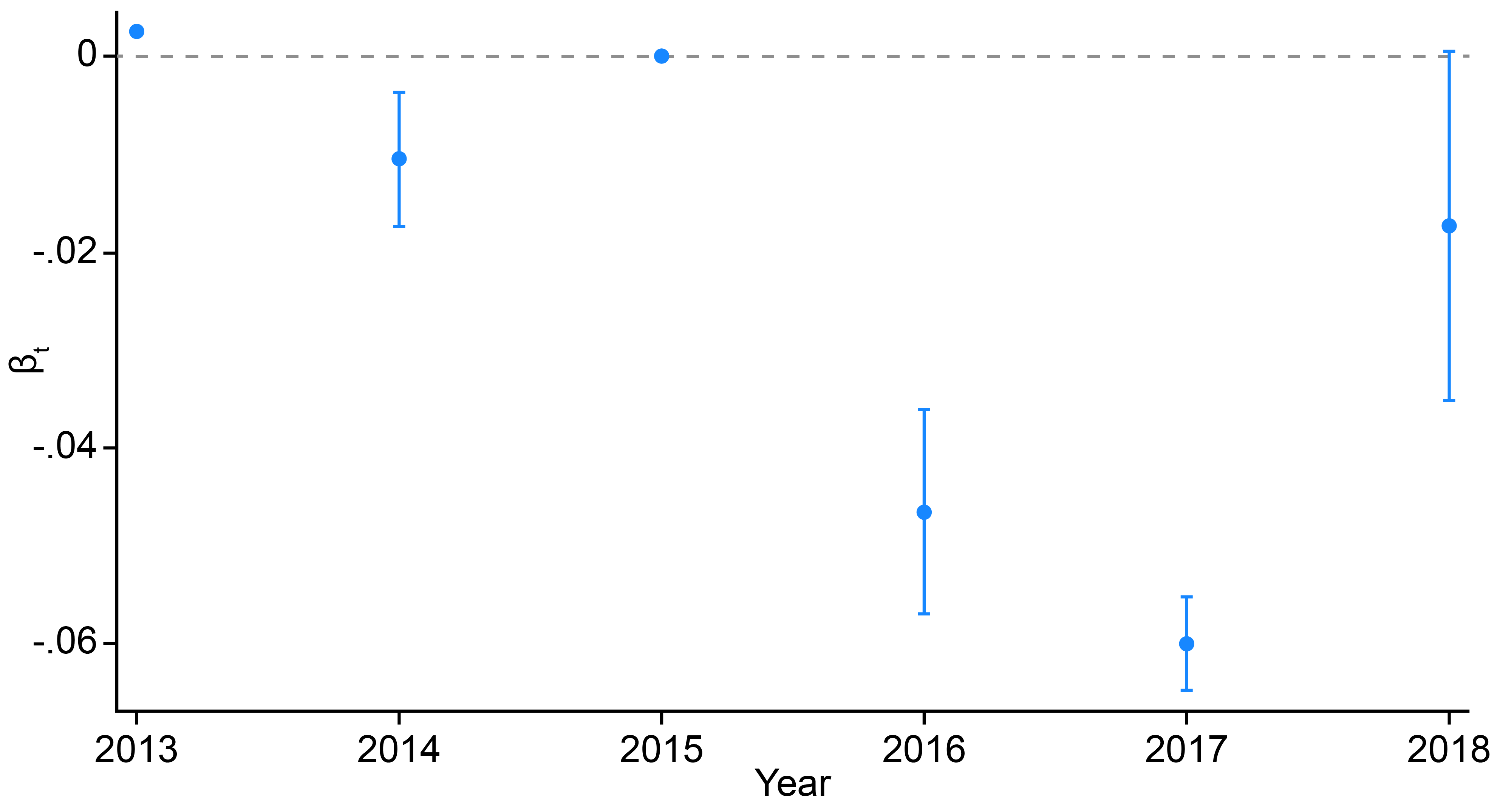

Supplement: Supplementary file 2 — Supplementary Fig 2. Event studies of difference-in-differences over time in all-cause mortality rate (TIF 168 KB) [file 44197_2023_181_MOESM2_ESM.tif]
