# Supplementary material for: A Quasi-experimental Study on the Effect of Pre-entry Tuberculosis Screening for Immigrants on Treatment Outcomes in South Korea: A Difference-in-Differences Analysis
Source: J Epidemiol Glob Health. 2024 Jan 23;14(1):154–61. doi: 10.1007/s44197-023-00181-6 (PMC11043236; doi:10.1007/s44197-023-00181-6)
Supplement: Supplementary file 3 — Supplementary file3 (DOCX 39 KB) [file 44197_2023_181_MOESM3_ESM.docx]

Supplemental Table 1 Characteristics of study population after propensity score matching (n=8,976)

| Variable | | | Korean (n=7,480) | | Immigrants (n=1,496) | |  |
| --- | --- | --- | --- | --- | --- | --- | --- |
|  |  |  | n | % | n | % | p-value |
| Age group | | ~14 | 13 | 0.17 | 3 | 0.2 | 1.000 |
|  | | 15-19 | 87 | 1.16 | 17 | 1.14 |  |
|  | | 20-24 | 580 | 7.75 | 116 | 7.75 |  |
|  | | 25-29 | 1,370 | 18.32 | 274 | 18.32 |  |
|  | | 30-34 | 910 | 12.17 | 182 | 12.17 |  |
|  | | 35-39 | 425 | 5.68 | 85 | 5.68 |  |
|  | | 40-44 | 560 | 7.49 | 112 | 7.49 |  |
|  | | 45-49 | 605 | 8.09 | 121 | 8.09 |  |
|  | | 50-54 | 780 | 10.43 | 156 | 10.43 |  |
|  | | 55-59 | 825 | 11.03 | 165 | 11.03 |  |
|  | | 60-64 | 565 | 7.55 | 113 | 7.55 |  |
|  | | 65-69 | 295 | 3.94 | 59 | 3.94 |  |
|  | | 70-74 | 140 | 1.87 | 28 | 1.87 |  |
|  | | 75~ | 325 | 4.34 | 65 | 4.34 |  |
|  | |  |  |  |  |  |  |
| Gender | | Male | 4,125 | 55.15 | 825 | 55.15 | 1.000 |
|  | | Female | 3,355 | 44.85 | 671 | 44.85 |  |
|  | |  |  |  |  |  |  |
| Disability | | None | 7,000 | 93.58 | 1,488 | 99.47 | <0.001 |
|  | | Mild | 277 | 3.7 | 2 | 0.13 |  |
|  | | Severe | 203 | 2.71 | 6 | 0.4 |  |
|  | |  |  |  |  |  |  |
| Income level | | Medical Aid | 15 | 0.2 | 3 | 0.2 | 1.000 |
|  | | 1^st^ quintile (lowest) | 1,560 | 20.86 | 312 | 20.86 |  |
|  | | 2^nd^ quintile | 1,997 | 26.7 | 399 | 26.67 |  |
|  | | 3^rd^ quintile | 3,078 | 41.15 | 616 | 41.18 |  |
|  | | 4^th^ quintile | 590 | 7.89 | 118 | 7.89 |  |
|  | | 5^th^ quintile (highest) | 240 | 3.21 | 48 | 3.21 |  |
|  | |  |  |  |  |  |  |
| Notification year | | 2013 | 1,850 | 24.73 | 370 | 24.73 | 1.000 |
|  | | 2014 | 2,110 | 28.21 | 422 | 28.21 |  |
|  | | 2015 | 2,240 | 29.95 | 448 | 29.95 |  |
|  | | 2016 | 210 | 2.81 | 42 | 2.81 |  |
|  | | 2017 | 750 | 10.03 | 150 | 10.03 |  |
|  | | 2018 | 320 | 4.28 | 64 | 4.28 |  |
|  | |  |  |  |  |  |  |
| Types of notification institution | | Public | 118 | 1.58 | 20 | 1.34 | 0.490 |
|  | | Private | 7,362 | 98.42 | 1,476 | 98.66 |  |
|  | |  |  |  |  |  |  |
| Smear test | | Negative | 5,360 | 71.66 | 1,124 | 75.13 | 0.006 |
|  | | Positive | 2,120 | 28.34 | 372 | 24.87 |  |
|  | |  |  |  |  |  |  |
| History of TB | | New case | 6,307 | 84.32 | 1,292 | 86.36 | 0.045 |
|  | | Relapse | 1,173 | 15.68 | 204 | 13.64 |  |
|  | |  |  |  |  |  |  |
| TB classification | | Pulmonary | 5,859 | 78.33 | 1,148 | 76.74 | 0.175 |
|  | | Extra pulmonary | 1,621 | 21.67 | 348 | 23.26 |  |
|  | |  |  |  |  |  |  |
| Comorbidities | |  |  |  |  |  |  |
|  | Malignancy | No | 7,382 | 98.69 | 1,477 | 98.73 | 0.901 |
|  |  | Yes | 98 | 1.31 | 19 | 1.27 |  |
|  |  |  |  |  |  |  |  |
|  | Kidney failure | No | 7,433 | 99.37 | 1,492 | 99.73 | 0.090 |
|  |  | Yes | 47 | 0.63 | 4 | 0.27 |  |
|  |  |  |  |  |  |  |  |
|  | Diabetes mellitus | No | 6,500 | 86.9 | 1,402 | 93.72 | <0.001 |
|  |  | Yes | 980 | 13.1 | 94 | 6.28 |  |

**Supplemental Table 2 Treatment success rate and all causes mortality rate of Korean and immigrants by year**

| Year | Korean | | Immigrants | | p-value |
| --- | --- | --- | --- | --- | --- |
|  | n | % | n | % |  |
| Treatment success rate (except for deaths during treatment) | | | | | |
| 2013 | 1,528 | 85.8 | 259 | 70.2 | <0.01 |
| 2014 | 1,793 | 87.9 | 306 | 73.2 | <0.01 |
| 2015 | 1,925 | 89.1 | 325 | 73.4 | <0.01 |
| 2016 | 173 | 85.6 | 32 | 80.0 | 0.37 |
| 2017 | 654 | 90.0 | 120 | 82.2 | 0.01 |
| 2018 | 278 | 90.0 | 56 | 87.5 | 0.56 |
| Total causes mortality rate | |  |  |  |  |
| 2013 | 215 | 11.6 | 15 | 4.1 | <0.01 |
| 2014 | 232 | 11.0 | 19 | 4.5 | <0.01 |
| 2015 | 210 | 9.4 | 9 | 2.0 | <0.01 |
| 2016 | 17 | 8.1 | 2 | 4.8 | 0.46 |
| 2017 | 54 | 7.2 | 9 | 6.0 | 0.60 |
| 2018 | 19 | 5.9 | 0 | 0.0 | 0.05 |

Table 3 Tuberculosis treatment success rate of Koreans and immigrants before and after the pre-entry tuberculosis screening at two different periods: (1) immediate policy effect without transition period, (2) 1 year transition period

|  |  | 1. Immediate policy effect | | | | | | 1. 1 year transition period | | | | | |
| --- | --- | --- | --- | --- | --- | --- | --- | --- | --- | --- | --- | --- | --- |
|  |  | Crude analysis | | | Adjusted model^a^ | | | Crude analysis | | | Adjusted model^a^ | | |
|  |  | Coef. | SE | p | Coef. | SE | p | Coef. | SE | p | Coef. | SE | p |
| Before policy | |  |  |  |  |  |  |  |  |  |  |  |  |
|  | Korean | 0.88 |  |  | 0.50 |  |  | 0.88 |  |  | 0.48 |  |  |
|  | Immigrants | 0.72 |  |  | 0.34 |  |  | 0.72 |  |  | 0.32 |  |  |
|  | Difference | -0.16 | 0.01 | <0.01 | -0.16 | 0.01 | <0.01 | -0.15 | 0.01 | <0.01 | -0.16 | 0.011 | <0.01 |
| After policy | |  |  |  |  |  |  |  |  |  |  |  |  |
|  | Korean | 0.92 |  |  | 0.53 |  |  | 0.89 |  |  | 0.50 |  |  |
|  | Immigrants | 0.81 |  |  | 0.43 |  |  | 0.85 |  |  | 0.45 |  |  |
|  | Difference | -0.105 | 0.02 | <0.01 | -0.10 | 0.02 | <0.01 | -0.05 | 0.028 | 0.088 | -0.05 | 0.027 | 0.085 |
|  |  |  |  |  |  |  |  |  |  |  |  |  |  |
| Difference-in-differences | | 0.05 | 0.02 | 0.037 | 0.06 | 0.02 | 0.014 | 0.11 | 0.03 | <0.01 | 0.11 | 0.029 | <0.01 |

^a^Covariates: gender, age, disability, household income, type of notification institution, result of smear, type of TB, history of TB, comorbidities (malignancy, kidney failure, diabetes mellitus)

Table 4 Mortality rate of Korean and immigrant tuberculosis patients before and after the pre-entry tuberculosis screening at two different periods: (1) immediate policy effect without transition period, (2) 1 year transition period

|  |  | 1. Immediate policy effect | | | | | | 1. 1 year transition period | | | | | |
| --- | --- | --- | --- | --- | --- | --- | --- | --- | --- | --- | --- | --- | --- |
|  |  | Crude analysis | | | Adjusted model^a^ | | | Crude analysis | | | Adjusted model^a^ | | |
|  |  | Coef. | SE | p | Coef. | SE | p | Coef. | SE | p | Coef. | SE | p |
| Before policy | |  |  |  |  |  |  |  |  |  |  |  |  |
|  | Korean | 0.12 |  |  | -0.11 |  |  | 0.11 |  |  | -0.10 |  |  |
|  | Immigrants | 0.04 |  |  | -0.18 |  |  | 0.04 |  |  | -0.16 |  |  |
|  | Difference | -0.08 | 0.01 | <0.01 | -0.06 | 0.01 | <0.01 | -0.07 | 0.01 | <0.01 | -0.06 | 0.01 | <0.01 |
| After policy | |  |  |  |  |  |  |  |  |  |  |  |  |
|  | Korean | 0.07 |  |  | -0.16 |  |  | 0.07 |  |  | -0.14 |  |  |
|  | Immigrants | 0.04 |  |  | -0.18 |  |  | 0.05 |  |  | -0.15 |  |  |
|  | Difference | -0.03 | 0.02 | 0.123 | -0.03 | 0.02 | 0.117 | -0.02 | 0.02 | 0.312 | -0.01 | 0.02 | 0.543 |
|  |  |  |  |  |  |  |  |  |  |  |  |  |  |
| Difference-in-differences | | 0.05 | 0.02 | 0.01 | 0.04 | 0.02 | 0.038 | 0.05 | 0.02 | 0.04 | 0.05 | 0.02 | 0.03 |

^a^Covariates: gender, age, disability, household income, type of notification institution, result of smear, type of TB, history of TB, comorbidities (malignancy, kidney failure, diabetes mellitus)
